# La recepción del freudomarxismo en la España del tardofranquismo y de la Transición, 1975-1978

**DOI:** 10.1590/S0104-59702023000100016

**Published:** 2023-05-15

**Authors:** Miguel Huertas-Maestro

**Affiliations:** i Investigador predoctoral, Instituto de Historia, Centro de Ciencias Humanas y Sociales/Consejo Superior de Investigaciones Científicas.Madrid – España miguel.huertas@csic.es

**Keywords:** psicoanálisis, marxismo, freudomarxismo, Transición, tardofranquismo, psychoanalysis, Marxism, Freudo-Marxism, Transition, late Francoism

## Abstract

Nuestro objetivo es mostrar el arraigo que tuvo el freudomarxismo, como forma específica de contacto entre marxismo y psicoanálisis, en la España del franquismo tardío y de la Transición (1975-1978). Analizamos la pertinencia del término “freudomarxismo”, sus diferencias con un psicoanálisis militante argentino influyente en corrientes sociales del psicoanálisis en España, y la revisión histórica del movimiento que realiza una figura relevante para la psicología española como es Antonio Caparrós i Benedicto. Finalmente abordamos la acogida relativa de la obra de Wilhelm Reich a través del esfuerzo de difusión de Ramón García y de la figura de Carlos Frigola, aprendiz de Eva Reich y creador de la Fundación Reich.

## Abstract (en)

Our objective is to show the roots of Freudo-Marxism as a specific form of contact between Marxism and psychoanalysis in the Spain of late Francoism and the Transition (1975-1978). We analyze the relevance of the term “Freudo-Marxism”, its differences with Argentine militant psychoanalysis influential in social currents of psychoanalysis in Spain, and the historical review of the movement carried out by a relevant figure for Spanish psychology such as Antonio Caparrós i Benedicto. Finally, we address the relative reception of the work of Wilhelm Reich through the dissemination effort of Ramón García and the figure of Carlos Frigola, Eva Reich’s apprentice and creator of the Reich Foundation.

Pese a que circuló de forma limitada, el psicoanálisis no fue prohibido durante la dictadura franquista (1939-1978). Estudios recientes ( Lévy, 2017 ) muestran cómo algunos conceptos de la teoría psicoanalítica, debidamente depurados y adaptados ideológicamente, encontraron su lugar en diversos ámbitos de la sociedad franquista tales como la criminología. Es notable la existencia de círculos de psicoanalistas en Madrid y Barcelona ( Carles et al., 2000 ); sin embargo, los pocos profesionales que los integraban ejercían con discreción, sin difundir sus ideas ni confrontar con la disciplina oficial del régimen ( Druet, 2017 ). En la década de los 1960, ya fuera de los ámbitos psicoanalíticos oficiales, sectores progresistas del ámbito psiquiátrico y del ámbito sociocultural, movilizados de forma activa contra el régimen, comenzaron a mostrar un interés creciente por las ideas psicoanalíticas, de lo que dan cuenta las publicaciones relacionadas tanto en revistas clínicas como en revistas culturales de izquierda (Druet, 2012a). Sin embargo, este interés no incluía los círculos oficiales de psicoanalistas, ya que su rigidez y jerarquía no eran del agrado de quienes buscaban un Freud con una dimensión política, con potencial subversivo, capaz de realizar un cuestionamiento crítico de la sociedad.

Es destacable el papel de los psicoanalistas del cono sur, fundamentalmente argentinos, que llegaron a España en la década de los 1970 como exiliados, proyectando una imagen diametralmente opuesta y de compromiso político, encontrando en el contexto de los últimos años del franquismo un suelo fértil que llevó a generar actividades, publicaciones, grupos de estudio y asociaciones (Druet, 2012b).

Este fenómeno ocurre en un contexto de intentos de mejora de la asistencia psiquiátrica desde una perspectiva crítica, comunitaria, y en pos de una mayor horizontalidad, tanto hacia los pacientes como entre los propios profesionales, lo que tomaría forma de huelgas, encierros y actos de solidaridad contestados con duras medidas administrativas y policiales, como dan cuenta los procesos de lugares como el Hospital Psiquiátrico de Asturias, las Clínicas de Ibiza 43 en Madrid, el Hospital Psiquiátrico de Salt o el Sanatorio Psiquiátrico de Conxo durante las llamadas luchas psiquiátricas del tardofranquismo ( Sáez Buenaventura, 1978 ; González de Chávez, 1980 ; Irisarri, 2017 ; Huertas, 2019 ).

La circulación de las ideas freudianas ha sido abordada en el ámbito latinoamericano por Ruperthuz (2017) para el caso de Chile y Plotkin (2003) para el argentino. Sus propuestas historiográficas han venido a renovar la manera de entender la historia del psicoanálisis, considerando éste como un producto cultural influido y modulado por el contexto histórico, social y político que se considere con independencia de la mayor o menos fidelidad de sus desarrollos a la ortodoxia marcada por las instituciones psicoanalíticas oficiales. En este sentido, la influencia de estos autores en la historiografía reciente, tanto en ámbitos latinoamericanos como europeos, es innegable ( Lévy, 2017 ). Una influencia que recoge también el presente artículo.

Para el caso español, la historiografía que ha estudiado la importancia de los psicoanalistas argentinos en esta última etapa de la dictadura que suele llamarse “tardofranquismo” (1969-1975) y la en la Transición (1975-1978) se han ocupado de la recepción del pensamiento lacaniano (Druet, 2012b; Averbach, Teszkiewicz, 2001; Bilbao, Huertas, 2019 ), prestando una menor atención al desarrollo de un pensamiento psicoanalítico con énfasis en lo social e influenciado por la teoría marxista.

El marxismo, no entendido aquí como la obra de Karl Marx sino como el cuerpo teórico-práctico de un movimiento político que, teniendo su origen en las obras de Marx y Engels, tuvo numerosos desarrollos, ha sido un agente determinante de la historia del siglo XX. Su influencia abarca desde el impacto en la academia hasta la generación de un movimiento revolucionario organizado en numerosos países. Su relación con el psicoanálisis, un diálogo no siempre fructífero, se cimenta en el cuestionamiento que ambas teorías hacen de los pilares de la civilización occidental, contándose tanto Marx como Freud entre los célebres maestros de la sospecha ( Ricoeur, 2007 ).

Ya desde principios de siglo podemos dar cuenta de las diferentes formas de este contacto. La primera enseñanza universitaria de psicoanálisis, por ejemplo, fue impartida por Sándor Ferenczi en 1919 durante el gobierno de Bela Kun en la breve República Soviética de Hungría ( Gutiérrez-Peláez, 2013 ). También puede mencionarse, a pesar del posterior desprecio de la psicología soviética hacia el campo psicodinámico, el acercamiento de Alexander Luria (1979) al psicoanálisis durante los años 1920. Asimismo, es relevante el Hogar Experimental de Niños, una casa pedagógica guiada por principios psicoanalíticos, establecida en Moscú entre 1921 y 1925, dirigida por Vera Schmidt con la participación de la analista rusa Sabina Spielrein y los psicólogos Alexander Luria y Lev Vygotsky ( Etkind, 1997 ; Falcón, nov. 2003). Por supuesto, es notable la influencia de Erich Fromm (1900-1980) en los desarrollos del psicoanálisis estadounidense a través de su obra, su práctica, y su actividad en el William Alanson White Institute ( Ávila, 2013 ). En relación a esto, autores como Pavón-Cuellar (2016) nos han brindado una relación histórica de carácter general entre marxismo y psicología.

En el contexto español, se ha abordado ya la manera en la que un sector politizado del psicoanálisis argentino encontró en la España del tardofranquismo y la en la Transición un medio fértil para sus aportaciones, que leían el psicoanálisis desde una óptica marxista, y que contribuirían a la formación de la perspectiva relacional del psicoanálisis en nuestro país ( Huertas-Maestro, 2021 ). Si bien la relación general entre el psicoanálisis y el marxismo no es el foco de este trabajo, sí pretendemos explicitar la manera en la que el contacto entre ambas teorías en la corriente freudomarxista tuvo cierto arraigo en la España de la época. Para ello, abordaremos la pertinencia del término “freudomarxismo” como denominación de esta corriente, algunas de sus diferencias con la perspectiva marxista del psicoanálisis, y la revisión histórica del movimiento que hace una figura de relevancia para la psicología española como es Antonio Caparrós i Benedicto. También las posiciones de José Bleger y Antonio Caparrós García-Moreno, autores que, junto a Marie Langer y otros analistas argentinos, ejercieron una indudable influencia en las corrientes más sociales del psicoanálisis en España. Finalmente, analizaremos la acogida relativa de la obra de Wilhelm Reich (1897-1957) a través del esfuerzo de difusión de Ramón García y de la figura de Carlos Frigola, aprendiz de Eva Reich y creador de la Fundación Reich.

## La misión freudomarxista

El nacimiento del freudomarxismo se ha localizado en una intersección entre la teoría psicoanalítica y el marxismo que se materializa, fundamentalmente, en las obras de Otto Fenichel, Siegfried Bernfeld, Erich Fromm y Wilhelm Reich ( Suárez, 1978 ). El contexto de su aparición es el tenso período de entreguerras que comprende el final de la Primera Guerra Mundial, la escisión del movimiento obrero en el ala socialdemócrata y los partidos comunistas, el avance de los últimos que acabaría por materializarse en la creación de la Unión Soviética, la crisis económica y social de 1929, y el auge del fascismo.

Para Suárez, las cuatro figuras principales del freudomarxismo aceptan el psicoanálisis sin reservas. Sin embargo, la asimilación teórica que hacen del mismo sería desigual. Algo similar ocurriría en su relación con el marxismo, al cual acceden a través de las concepciones de una segunda Internacional, hegemónica desde el punto de vista ideológico en su momento, pero abandonada por sus elementos revolucionarios – los que formarían el movimiento comunista –, quienes la juzgan lastrada por el positivismo, el mecanicismo, el economicismo y el voluntarismo (Suárez, 1978, p.141). En adición a una comprensión relativamente limitada de las teorías a combinar, las propuestas freudomarxistas se estrellarían con la desconfianza, la oposición o la incomprensión de las organizaciones marxistas y las psicoanalíticas. Wilhelm Reich sería expulsado tanto del Partido Comunista como de la Asociación Psicoanalítica Vienesa; tras asentarse en Nueva York en 1939, donde el desarrollo de su línea de investigación más biológica y energética le lleva a construir “acumuladores de orgón” que absorberían parte de la energía vita de los organismos con efectos beneficiosos para la salud tales como la cura del cáncer. En 1947 y a instancias de la Administración de Alimentos y Medicamentos, la venta de estos acumuladores es considerada estafa y, en 1956, Reich llega a ser condenado a dos años de cárcel por violar esta sentencia, muriendo en la cárcel un año más tarde. Por su parte, Otto Fenichel y Siegfried Bernfeld, aunque continúan con su actividad dentro de las instituciones oficiales de psicoanálisis, abandonan el marxismo; Erich Fromm, quien se mostraba crítico con los Estados socialistas, hace su propio camino a través del desarrollo de una perspectiva social y cultural del psicoanálisis, lo que le lleva a fundar el William Alanson White Institute en Nueva York junto con Clara Thompson, Harry Stack Sullivan, Frieda Fromm-Reichmann y David y Janet Rioch.

Fromm sería, quizá, el más influyente de ellos, generando una perspectiva psicoanalítica crítica con la teoría pulsional – según la cual la base de la psique son impulsos internos que buscan liberar tensión energética a través de objetos no predeterminados – y la universalidad del complejo de Edipo, y con énfasis en la interrelación entre persona y sociedad. De una práctica ortodoxa del judaísmo y el estudio de los textos sagrados, se orienta posteriormente hacia el psicoanálisis de la mano de la que sería su esposa, Frieda Reichmann, tomando contacto con psicoanalistas de tendencias socialistas como Bernfeld o Reich mientras se encarga del programa del Instituto de Investigación Social, institución en la que Max Horkheimer desea introducir el psicoanálisis con un propósito interdisciplinar. Fromm participaría en este organismo hasta 1939, momento en que también abandona la institución, entre otros, Walter Benjamin ( Castaño, 2013 , p.164). El materialismo histórico marxista tendría, desde su perspectiva, un mayor peso explicativo que el materialismo fisiologicista freudiano, idea que sostendría después de su emigración a América:

La contribución más duradera e importante de Marx acerca de las leyes que gobiernan a la sociedad parte de la premisa siguiente: antes de que el hombre pueda dedicarse a cualquier tipo de actividad cultural, tiene que producir los medios para su subsistencia física. … Entendía los fenómenos espirituales como producidos por fenómenos materiales. Basaba su teoría en la actividad del hombre y no en la fisiología del hombre ( Fromm, 1974 , p.217-218).

Esta lógica marxista sería una influencia, entre otras, para sus propuestas teóricas y técnicas. Hace énfasis en la dimensión social – lenguaje, lógica, costumbres – de las dinámicas de lo inconsciente y lo reprimido, considerando también la existencia de rasgos de carácter social determinadas por las formas de producción y el estilo de vida de las clases sociales de una sociedad concreta; también adaptaría la técnica clásica hacia procedimientos más directos y con mayor participación del analista ( Castaño, 2013 ).

Tanto el movimiento psicoanalítico como el comunista comparten ciertas similitudes, las cuales sirven tanto de cimento lógico del freudomarxismo como de justificación de su labor. Mientras que el marxismo revolucionario siempre abogó por combinar el arma de la crítica con la crítica de las armas (Marx, 2005), el psicoanálisis se pronuncia críticamente sobre una sociedad que percibe monolítica e incapaz de transformar ( Castilla del Pino, 1971 , p.56). Con todo, ambas teorías se imponen objetivos desmitificadores – de ilusiones, de la falsa conciencia – y también emancipadores – del neurótico, del proletariado –, y para lograrlos el sujeto debe tomar conciencia de aquello que lo condiciona. Ambas teorías recurren a modelos explicativos de carácter tópico: encontramos, por un lado, el Preconsciente-Consciente e Inconsciente de la primera tópica freudiana, y por otro la infraestructura productiva y la superestructura ideológica propia del análisis marxista. Encontramos asimismo explicaciones dinámicas en el conflicto pulsional y en la lucha de clases. Además de lo anterior, los freudomarxistas hallan una potencial compenetración entre ambas teorías. Un movimiento comunista triunfante garantizaría unas condiciones de vida adecuadas y la posibilidad del desarrollo personal, así como la transformación de estructuras familiares patógenas. El psicoanálisis, por su parte, sería capaz de arrojar luz sobre la problemática sexual y la psicopatología derivada de la miseria económica y la opresión de las instituciones burguesas, convirtiéndose así en una suerte de conciencia auxiliar al servicio del movimiento obrero ( Suárez, 1978 ).

Si bien el freudomarxismo no cristalizaría de la forma que sus precursores pretendían, se ha considerado un precursor de la crítica de las prácticas, valores y consensos culturales acerca de la vida sexual e interpersonal que posteriormente se ligaría al feminismo, al movimiento juvenil y a la lucha por la emancipación sexual que se produjo durante la segunda mitad del siglo XX. Pavón-Cuellar (2017) encuentra esta relación en Otto Fenichel, Siegfried Bernfeld y, especialmente, en Wilhelm Reich. De este modo, el foco en lo subjetivo y en una vida psíquica con potencial político se considera precedente de la importancia de lo personal y lo micropolítico en los movimientos sociales del último tercio del siglo XX.

En España, uno de los autores que más precozmente abordan, en el ámbito académico, el freudomarxismo, es Antonio Caparrós i Benedicto (1938-2001). Licenciado en teología y miembro de la Compañía de Jesús hasta 1968, se familiariza con la obra de Freud y el pensamiento del analista Víctor Frankl durante estancias que, por sus estudios teológicos, realiza en Austria y Viena. Llega a ser catedrático de psicología en la Universidad de Barcelona, rector de la misma durante ocho años – entre 1994 y 2001 – y miembro fundador de la Sociedad Española de Historia de la Psicología ( Siguan, 2002 ). También reflexiona sobre las relaciones entre psicoanálisis y marxismo ( Anguera, 2002 ). Su tesis doctoral, de 1974, versa sobre *El carácter social según Erich Fromm* , publicada como libro un año más tarde (Caparrós i Benedicto, 1975a); también en 1975, publica un interesante artículo en el que ofrece un balance histórico del freudomarxismo. Como veremos más adelante, Caparrós i Benedicto entiende, en una línea similar a la sostenida por José Bleger (1922-1972), que ambas teorías no pueden hermanarse en el mismo nivel explicativo solo por el hecho de ser herramientas de crítica. Sin embargo, y pese a diferenciarse de dicha nomenclatura, no parece entender el término como algo negativo en sí mismo y, de hecho, afirma que ha generado resultados de interés al profundizar en ciertos aspectos tanto del marxismo como del psicoanálisis, además de recuperar publicaciones de autores de importancia como un “casi olvidado” Wilhelm Reich, Bernfeld o el primer Fromm (Caparrós i Benedicto, 1975b, p.5). Tras analizar las propuestas de estos autores, y pese a su heterogeneidad, concluye que sí puede hablarse de una unidad de principios y un punto de partida común que permite hablar del freudomarxismo como una corriente cohesiva y diferenciada del resto. También es crítico: el error a la hora de sintetizar ambas teorías obedece al intento de justificar la naturaleza dialéctica e histórica del psicoanálisis ante sus detractores en el campo del marxismo. “La argumentación – y este es otro de los graves errores freudomarxistas – es puramente formal y abstracta … ¿marxistas y psicoanalistas entienden lo mismo cuando hablan de idealista, dialéctico, histórico etc.?” (p.30).

Más allá de los equívocos nominalistas, lo central para Caparrós i Benedicto es que permanecen en ese nivel semántico y renuncian a profundizar en estos debates, perdiéndose así la oportunidad de ahondar en la posible convergencia entre las dos teorías que nos ocupan.

Por otra parte, y por mucho que se adjetive al psicoanálisis con palabras afines al marxismo dominante de la época – como ya hemos visto: dialéctico, materialista, histórico –, existen ciertas diferencias de principios que no podrían reconciliarse. Tenemos, así, la noción de un inconsciente que surge al margen de la realidad social y la diametralmente opuesta teoría leninista del reflejo, según la cual toda sensación emana del mundo objetivo.

La propuesta freudomarxista se basaría en entregar a la revolución un psicoanálisis debidamente depurado de idealismo e ideología burguesa, para que así pueda operar como herramienta explicativa tanto de la formación de superestructura ideológica como del comportamiento de las masas, que estarían aún bajo el influjo de un superyó condicionado por estructuras sociales “todavía burguesas”, y que no habría llegado aún a transformarse según las nuevas condiciones económico-sociales traídas por la revolución. Cabe destacar que Caparrós i Benedicto no desprecia, en absoluto, el posible valor de este trabajo, pero sí lo considera estéril cuando su formulación es “puramente formal” (Caparrós i Benedicto, 1975b, p.31). Al intentar que ambas teorías converjan, recurren al modelo base-superestructura del marxismo, introduciendo las pulsiones freudianas junto a la base económica como cimento fundamental de la sociedad. De esta forma, se diluye tanto el conflicto pulsional como la realidad sociocultural de la propuesta psicoanalítica. También queda desvirtuado el marxismo, ya que la infraestructura de una sociedad ya no está compuesta, únicamente, por las relaciones sociales de producción. La cuestión estaría clara que “al pretender, entonces, insertar el psicoanálisis dentro del sistema filosófico marxista acaban por desvirtuar al uno y al otro” (Caparrós i Benedicto, 1975b, p.31). Los argumentos freudomarxistas resultaron insuficientes a la hora de convencer a la *intelligentsia* soviética.

A pesar de señalar críticamente los errores en los que, a su juicio, incurre el freudomarxismo, la visión de Caparrós i Benedicto es que estos solo pueden señalarse con la perspectiva que da el paso del tiempo. Para él, quien busque profundizar en la posible relación entre psicoanálisis y marxismo debe encontrar un puente en “el hecho de que ambos tienen un objeto común, que no es otro que los individuos socializados en una forma histórico-social concreta” (p.32).

## El psicoanálisis militante

A pesar de estas reflexiones en torno al freudomarxismo realizadas en determinados nichos académicos, una parte importante de la recepción del psicoanálisis social en la España de los años 1970 y 1980 debemos buscarla en las ideas y prácticas procedentes del importante núcleo de producción de conocimiento psicoanalítico que representa Argentina. En este ámbito, la denominación de esta corriente como “freudomarxista” ha sido discutida por autores como Vainer (oct. 2003), quien afirma que “el freudomarxismo nunca existió”. El término sería, entonces, un descalificativo que etiquetaría a un grupo heterogéneo de analistas que, enmarcados en coordenadas ideológicas de izquierda, buscaron algunos puntos de unión entre ambas teorías “sin pretensión de totalidad”. Según Vanier, una figura importante para el contexto argentino como José Bleger – quien fundaría en 1954 la Asociación Psicoanalítica Argentina junto a Enrique Pichón Rivière, Marie Langer, León Grinberg, Arnaldo Rascovsky y el español exiliado Ángel Garma, entre otros – habría matizado sus propias posiciones, alejándose del término, por ser dañino para su reputación. Si bien es cierto que Bleger (1972) realiza un ejercicio de precisión teórica en “Psicoanálisis y marxismo”, donde diferencia el freudomarxismo de sus propias posiciones, no vemos aquí un intento de alejarse de una etiqueta peligrosa, sino un ejercicio militante que sigue la lógica de su anterior y polémico trabajo *Psicoanálisis y dialéctica materialista* ( Bleger, 1958 ), por el que sería expulsado del Partido Comunista de Argentina ( Dagfal, 2010 ; Vezzetti, 2016 ). Para Bleger, el término “freudomarxismo” nombra una fusión de ambas teorías en pie de igualdad, una concepción en la que ambas se consideran capaces de formar una visión general del mundo, una cosmovisión. Considera este camino un error, siendo su propuesta comprender las ciencias concretas – entre ellas, el psicoanálisis – desde una cosmovisión marxista: “No se trata tampoco en nuestros estudios de traer la dialéctica desde afuera por referencia a otros campos del conocimiento; se trata de que la dialéctica ya está en los hechos y son éstos los que exigen su introducción consecuente en la teoría como forma de hacer más lúcida la experiencia misma” (Bleger, 1958, p.19).

El pensamiento de Bleger sería muy influido por los trabajos de Georges Politzer, filósofo húngaro-francés, militante comunista y miembro de la resistencia francesa contra los nazis – por lo que sería fusilado durante la Segunda Guerra Mundial –, quien realiza una crítica al psicoanálisis durante los años 1920, en la que argumenta que la lucidez de la teoría freudiana y la innovación que representa se ve lastrada por el biologicismo, el mecanicismo y cierto espiritualismo. Para Bleger, esta sería una de las críticas más interesantes al psicoanálisis ( Liberman, 2013 ). Bleger no pretende oponerse al psicoanálisis, sino contribuir a su desarrollo crítico. Dado que el psicoanálisis es una ciencia concreta y el marxismo una cosmovisión, situar ambos al mismo nivel para así combinarlos requiere reducir el marxismo a su faceta económica o bien emplear un concepto demasiado amplio de psicoanálisis: por ejemplo, al considerar los fenómenos psicológicos como una explicación de los conflictos sociales o económicos (Bleger, 1972). Con esto, Bleger considera estar separando sus propias posiciones de las del freudomarxismo, movimiento que eleva el psicoanálisis a la categoría de cosmovisión para poder integrar ambas teorías.

Otra figura de relevancia en el medio argentino, que ejercería una notable influencia en algunos círculos psicoanalíticos españoles, es Antonio Caparrós García-Moreno (1927-1986), que no debe confundirse con el ya citado Antonio Caparrós i Benedicto a pesar de la coincidencia de nombre y primer apellido. Analista de origen madrileño, Caparrós García-Moreno cursa sus estudios de medicina, psiquiatría y psicoanálisis en Argentina, donde sería profesor titular de la cátedra de Psicología General de la Universidad de Buenos Aires durante los años 1960, puesto que volvería a ocupar entre 1973 y 1976, momento en el que regresa a España como consecuencia de una grave persecución política tras el golpe de Estado y la instauración de la dictadura militar en Argentina. Hace trabajo militante en Cuba en 1969, como “gestor del programa de educación de la Revolución” (García de la Hoz, 1987, p.42), viviendo de cerca tanto los movimientos revolucionarios latinoamericanos – fue amigo personal de Ernesto Guevara – como la crisis del psicoanálisis argentino ( Ávila-Espada, 1987 ).

Las posiciones de Caparrós también son influidas por la obra de Politzer, y trataría de adaptar sus propuestas a su contexto social e histórico. Para ello enfoca críticamente las diferencias entre la teoría y la práctica realmente existente, la adopción de modelos psicológicos desarrollados en otros contextos (por ejemplo, en los países de capitalismo desarrollado), y la diferenciación entre teorías científicas y el uso ideológico de las mismas. También realiza críticas a la técnica psicoanalítica tradicional, como por ejemplo el uso autoritario del encuadre. Su propuesta iría en sintonía con la “psicología concreta” de Politzer: desarrollar una teoría y técnica que tenga como concepto central que la persona no es sino la sociedad individualizada ( Ávila-Espada, 1987 ).

En 1987, la revista *Clínica y Análisis Grupal* dedica, con motivo de su fallecimiento, un número homenaje a Antonio Caparrós García-Moreno que cuenta con textos de su primo Nicolás Caparrós, así como de Alejandro Ávila, Armando Bauleo, Hernán Kesselman y Antonio García de la Hoz (1987). Artículos en los que se analiza su figura y se reconoce la notable influencia que su pensamiento ejerció en la construcción de un psicoanálisis social en la España de los 1970. En uno de ellos, precisamente el que analiza la influencia de la obra de Politzer en el pensamiento de Caparrós García-Moreno, se señala cómo éste era también crítico con el freudomarxismo, al que consideraba, pese a sus aciertos, un emergente de las contradicciones sociales de la época:

En mi caso personal lo que no me parecía adecuado es etiquetarme de algo así como freudomarxista o de psicoanálisis marxista o cualquiera de estos eclecticismos que se han pretendido realizar y que retiradamente se llevan a cabo, cíclicamente. El psicoanálisis ha sido y es un aporte fundamental que cambió todo el modo de ver sobre el comportamiento del hombre, el hombre mismo, y desde luego todos los aspectos psicológicos. No es solamente el descubrimiento del inconsciente, que se puede dudar de si fue Freud mismo o si fue anterior a él, sino toda la teoría analítica. Ahora bien, la teoría analítica parte de un momento histórico determinado: Los finales de la belle époque de la burguesía, en Viena, y de unas capas medias que no conseguían ya lograr lo que, el sistema les proponía, al mismo tiempo que se daban cambios de valores y entonces no podían aferrarse a esos valores para mantenerse en ellos. Todo ello llevaba a que, además de sus descubrimientos esenciales, Freud – y es lógico, él no podía hacer otra cosa – tuvo que crear una teoría psicológica que llenase esta necesidad de la época. Insisto, como muchos aciertos y verdades científicas, pero al mismo tiempo con el lema del gatopardismo de cambiar para que nada cambie. La sociedad se desdibujaba, lo fundamental era instintivo, había fases y símbolos universales etc. Yo he aprendido casi todo lo que sé de psicología de la psicología freudiana, con algunas salvedades: No se trata del individuo *versus* la sociedad, sino que el individuo es el emergente, la personificación de la sociedad. Y sin conocer todos esos aspectos que son la estructura social, los avatares de la historia personal y los modos en que todo ello se hace psicología de la personalidad en un hombre, es imposible comprender nada de ese individuo (citado en Ávila-Espada, 1987 ).

La influencia de Caparrós García-Moreno y de otros autores situados en esta línea de pensamiento fue muy notable en los desarrollos teóricos del llamado psicoanálisis relacional en España. Años más tarde, destacados representantes del psicoanálisis relacional, se reconocen

herederos no designados del espíritu cuestionador y libre de Ferenczi, del ansia transformadora de Rank, del inconformismo social de Fenichel, del cuestionamiento Reichiano al Freudismo, de los pensadores de la Escuela de Frankfurt, del freudo-marxismo, la Psicología concreta de Politzer, y sus emergentes latinoamericanos Marie Langer, José Bleger, Antonio Caparrós, y su portavoz en España, Nicolás Caparrós. Ellos y nosotros, fuimos fecundados directa o indirectamente con el amplio horizonte que Enrique Pichón Rivière le dio al Psicoanálisis al releerlo como Psicología Social ( Ávila-Espada et al., 2007 , p.129).

Como queda reflejado, el interés por la obra de Fromm y su fértil trayectoria sería recogida por psicoanalistas orientados hacia una perspectiva social, mientras que la figura de Wilhelm Reich, también de marcado componente social y político pero muy orientada a lo fisiológico, tendría también su área de recepción en territorio español.

## La recepción de Wilhelm Reich

Como ya hemos mencionado, una figura como la de Wilhelm Reich era de indudable interés para quienes buscaban el potencial político en el estudio de la subjetividad. Sin embargo, Reich fallecería en 1957 y, tal y como nos relata Carlos Frigola (jul. 2015, p.24-25),

dejó escrito en su testamento que sus trabajos originales no podrían ser estudiados hasta 50 años después de su muerte, es decir hasta el año 2007. Desde entonces la editorial de Nueva York Farrar, Straus y Giroux está publicando la mayoría de sus escritos inéditos. En España, el desconocimiento de la obra de Reich es total. Tenemos que tener en cuenta que en nuestro país solo se ha publicado el 10% de su obra en lengua castellana, y la mayoría de las publicaciones solo en Sudamérica.

Ramón García, uno de los psiquiatras más representativos de los movimientos críticos de los años 1970, es quien comienza la divulgación de su obra en España. García ya había participado en varias redes de carácter progresista, como la corriente crítica de estudiantes, psiquiatras y psicopedagogos que funciona desde el 1967 hasta el 1973, o el Grupo de Psiquiatras Progresistas, a cuyas reuniones acuden figuras como Castilla del Pino, Tosquelles o el propio Franco Basaglia en 1971; también impulsa la creación del Colectivo Universitario de Antipsiquiatría ( Irisarri, 2017 , p.157) y participa en la Coordinadora Psiquiátrica ( Huertas, 2017 ). Duro en su crítica manicomial ( García, 1979 ) y también con la reforma psiquiátrica ( García, 1995 ), García comparte posiciones tanto con la antipsiquiatría anglosajona como con la psiquiatría crítica italiana. Prologa la primera edición en castellano de *La institución negada* de Basaglia (García, Serós, Torrent, 1972); lleva a cabo una labor de difusión de un discurso crítico en psiquiatría en obras como *¿Psiquiatría o ideología de la locura?* (1972), donde publica un texto suyo junto a otros de Franco Basaglia y Franca Basaglia Ognaro (García, Basaglia, Basaglia, 1972); y en *Psiquiatría, antipsiquiatría y orden manicomial* , García (1975a) recompila textos de Basaglia, Carrino, Castel, Espinosa, Castella y Casagrande. Su interés por lo psicodinámico ya le había llevado a publicar *Freud y el psicoanálisis* (Bosch, García, Sarró, 1973).

El interés de Ramón García por divulgar la obra de Wilhelm Reich se plasma en uno de los libros de la colección que dirigía en Anagrama, *Psicoanálisis y sociedad: apuntes de freudo-marxismo* (García, 1975b), una serie de dos volúmenes que pretendía ilustrar dos variantes de la relación entre marxismo y psicoanálisis, siendo la primera Wilhelm Reich y la segunda Igor Caruso, éste último un freudiano más estricto y fundador también del Círculo Vienés de Psicología Profunda en 1947 (García, 1975b, p.7). Respecto a Wilhelm Reich, a quien García considera un desconocido que empieza a no serlo tanto, presenta su texto de 1934 “La aplicación del psicoanálisis a la investigación histórica”, añadiendo también unas notas en su apéndice que ilustran fragmentos importantes de las célebres obras de Reich *Materialismo dialéctico y psicoanálisis* – original de 1934 y ya publicada en castellano por la editorial Siglo XXI en México (Reich, 1972c) – y *Psicología de masas del fascismo,* trabajos de muy difícil acceso en la época (García, 1975b, p.8). Además, introduce estos trabajos con su propio texto “Contribución a la obra de Wilhelm Reich”, el cual

no quiere ser más de lo que el título expresa: un aporte de información para el estudio de Reich. Si introducir es, tal como pienso, plantearse la crítica de aquello a lo que se introduce, mi trabajo no debe ser considerado una introducción a la obra de Reich; tal introducción queda, así, pendiente de posibles futuros esfuerzos. Nuestra pretensión ha sido, simplemente, sistematizar y resumir la biografía de Reich, orientar sobre los hilos temáticos fundamentales de su obra y aportar un amplio material bibliográfico suficientemente trabajado (García, 1975b, p.8).

Así pues, en primer lugar, García expone una síntesis de la biografía de Reich, agrupada por años. Tras esto, la exposición sobre la bibliografía reichiana se basa, esencialmente, en una selección de *L’oeuvre de Wilhelm Reich* de Constantin Sinelnikoff (1970) , a la que considera de gran precisión y admite no haber modificado más que unos pocos detalles (García, 1975b, p.61). El trabajo de García trata de establecer relaciones entre lo ofrecido por Sinelnikoff y las publicaciones de Reich disponibles en castellano y algunas nuevas ediciones de la época, formando así un apartado bibliográfico, ordenado cronológicamente, en el que se indica el título – en alemán o en inglés – del texto, su año de edición, y su posible traducción al castellano, así como ediciones posteriores o ediciones anónimas, admitiendo en este caso que se trata de un trabajo incompleto (García, 1975b, p.62). En un segundo apartado recogen escritos de Reich traducidos al castellano, una sección mucho más reducida que cuenta con ediciones argentinas, mexicanas, y francesas. Las únicas dos publicaciones desde el territorio español son, precisamente, publicadas por Anagrama. La primera es *Reich habla de Freud* , una entrevista en la que Reich (1970) conversa con un representante de los Sigmund Freud Archives, publicada por Anagrama en su colección Argumentos en 1970 junto con una selección de textos de Reich y parte de su correspondencia inédita. La segunda es “Los padres como educadores: la compulsión a educar y sus causas”, de mediados de los años 1920, incluida en el volumen *Psicoanálisis y educación* de la colección Cuadernos de Anagrama ( Reich, 1973 ).

El tercer apartado se refiere a trabajos dedicados íntegralmente a Reich, entre los que destacan los publicados por los Grupos de Trabajo de Psicología Crítica en 1971 (García, 1975b, p.88). Por último, García añade los “comentarios a Reich”: obras que abordan su figura o parte de su obra siendo otro su foco principal de estudio. Aquí tenemos obras como *La personalidad autoritaria* , de Adorno (1965) ; el trabajo *Psicoanálisis y dialéctica materialista* , del argentino José Bleger (1958) ; los *Escritos* , de Jacques Lacan (1966) ; el intento de relación entre psicoanálisis y marxismo que hace Carlos Castilla del Pino (1971) en *Psicoanálisis y marxismo* ; *Eros y civilización* , de Marcuse (1965) ; *Vigencia de Sigmund Freud* , de Pontalis (1971) ; o la obra de Ronald Laing (1972)*Esquizofrenia y presión social* .

Para Ramón García, Reich representa “la apertura a lo social de la cosa psiquiátrica”, además de un ejemplo de praxis: “Había creado además cosas que se parecía mucho a lo que nosotros nos gustaría plantearnos en barrios, en comarcas en relación con la cosa psiquiátrica abierta al barrio, a la gente y no exclusivamente tecnificada psiquiátricamente” ( Irisarri, 2017 , p.399).

Por su parte, Carlos Frigola es uno de los representantes de mayor relevancia de la escuela reichiana en la España de la época. Tal y como analiza Fabiola Irisarri (2017) , después de estudiar psiquiatría con Ramón Sarró, viaja a Londres en 1972 para formarse como psicoanalista gracias a una beca del Tavistock Institute. Allí, también participa y se forma en las comunidades terapéuticas de Philadephia Association, dentro del movimiento antipsiquiátrico británico. Allí conoce a Ronald Laing, pero es con Aaron Esterson con quien traba una relación más estrecha. En este período, Frigola entra en contacto con Eva Reich, la hija de Wilhelm, figura con la que se forma y analiza. Esta doble vía, la de la comunidad terapéutica y la de la terapéutica reichiana, no es para Frigola una coincidencia: la relación que Wilhelm Reich establece entre represión sexual y opresión política, al igual que su propuesta de relacionar atmósfera y psique, permite pensar desde una perspectiva global del mundo, una visión que comparte una clara vocación emancipadora con las comunidades terapéuticas impulsadas desde la antipsiquiatría, como Kingsley Hall o las agrupadas en la Philadephia Association.

En 1977, y ya en España, Frigola abre en Creixell, Cataluña, la comunidad terapéutica Existentialia junto a Pilar Castro, y funda también la Fundación Wilhelm Reich. Merece la pena señalar que esta última se concibe también como un centro de planificación familiar por petición expresa de Eva Reich ( Irisarri, 2017 , p.168). Existentialia, que funciona durante algo más de dos años, busca servir como lugar seguro en situaciones de crisis; además, se aplica el psicoanálisis reichiano. La Fundación Wilhelm Reich sigue abierta en la actualidad, si bien funciona como una comunidad terapéutica de día.

Frigola, que parte de “la concepción de la locura como un todo global” ( Irisarri, 2017 , p.169), se uniría a finales de los años 1970 al colectivo francés SexPol – llamado así en honor de la plataforma fundada por Wilhelm Reich en el período de entreguerras y cuyo nombre completo sería “Asociación alemana para una Política Sexual Proletaria” –, y cuyo objetivo sería enlazar política y sexualidad.

Lo social, lo biológico y lo psíquico quedan ligados por la concepción reichiana. La patología, tanto a nivel individual como a nivel social, se relaciona con el grado de represión exigido por la cultura. La faceta biológica queda cubierta con la propuesta del orgón como una partícula sexual que podría ser medida y manipulada de forma experimental. La energía orgónica se ve modificada por presiones internas y externas sobre el individuo y, de esta forma, la represión podría ser medida en términos físicos (Reich, 1972a). Al intentar conectar los mecanismos explicativos de las ciencias positivas con los de la crítica a la sociedad, el papel de profesional experto adquiere una magnitud política además de la terapéutica ( Irisarri, 2017 , p.170). *La lucha sexual de los jóvenes* , publicado originalmente en 1932, es, probablemente, la obra de Reich más difundida en la época que nos ocupa, en especial sus ediciones argentina y mexicana (Reich, 1972b, 1974); la influencia del freudomarxismo y la Sexualpolitik de Reich en la llamada reforma sexual ha sido analizada por sexólogos españoles como Efigenio Amezúa (2004) , quien también sería miembro fundador de la Sociedad Española de Sexología.

En la actualidad existe a nivel estatal español una Fundación Sexpol, estabelecida en los ochenta pero que tiene su origen en 1979 con la formación de la Sociedad Sexológica de Madrid de la mano de sociedades similares en otras regiones, con una vocación educativa, divulgativa y terapéutica respecto al malestar sexual de la sociedad. Una revista bimensual de divulgación, *Sexpol* , permanece en activo desde 1981. Pese al tributo reichiano de su nombre, la Fundación Sexpol explicita que no mantiene en la actualidad ni la línea de Reich ni ninguna orientación psicodinámica, apoyándose para sus tratamientos en estrategias cognitivo-conductuales (Fundación Sexpol, s.f.).

Wilhelm Reich sí seguirá siendo una figura fundamental de interés para Frigola, quien publica, ya en 2015, dos artículos (Frigola, ene. 2015, jul. 2015) que analizan su legado atendiendo a la primera mitad de los años 1930 y, en especial, a la celebración en Suiza del Congreso de Lucerna en 1934 – el 13º Congreso Psicoanalítico Internacional –, el primero en ser celebrado con Adolf Hitler como canciller de Alemania y en el que confluyeron diversos fenómenos de importancia para el movimiento psicoanalítico (Frigola, jul. 2015). Lo primero, las relaciones entre las instituciones psicoanalíticas alemana y austríaca y el régimen nazi. En 1933, el año anterior, se decreta que los profesionales judíos deben abandonar las profesiones sanitarias. Ante esto, la oficialidad psicoanalítica reacciona de forma ambivalente, en ciertos casos pidiendo la baja voluntaria de sus miembros judíos, como ocurre en el caso de Max Eitingon. Merece la pena destacar que el Instituto Psicoanalítico de Berlín sería incorporado pocos años más tarde al Instituto Alemán para la Investigación Psicológica y Psicoterapéutica, presidida por el primo del Hermann Göring, momento a partir del cual gran parte de los psicoanalistas no judíos irían goteando fuera de la institución (Frigola, jul. 2015). El Congreso de Lucerna presenta, así, un antes y un después en la historia del psicoanálisis al ser el primer congreso en el que la mayoría de los analistas de origen judío ya había emigrado o estaba preparándose para hacerlo.

La controversia de la aparición de la pulsión de muerte en la teoría del joven movimiento psicoanalítico es el segundo fenómeno en ser abordado por Frigola. Este nuevo concepto, pese a que tenía el objetivo de solventar problemas clínicos aún sin explicación – entre ellos, el masoquismo y las neurosis de guerra –, no encaja sin resistencia. Una polémica de especial relevancia emerge a partir de los desarrollos teóricos de Sandor Ferenczi, a quien Freud ya pidió que no presentase, en el Congreso de Wiesbaden de 1932, su trabajo “Confusión de lenguas entre el adulto y el niño: el lenguaje de la ternura y el lenguaje de la pasión”, que no vería la luz hasta ser rescatado por Balint en 1949 ( Coderch, 2014 ). En este texto, Ferenczi analiza el daño que deriva de las situaciones de abuso infantil y explica la dinámica por la cual el adulto, por su condición patológica, interpreta el lenguaje tierno del niño como pasión sexual, produciéndose así el abuso que perturbará su desarrollo (Ferenczi, 1984a). La controversia vendría del énfasis de Ferenczi en el factor externo, que a su juicio habría quedado relegado en favor de explicaciones relativas a la constitución y la predisposición (Ferenczi, 1984b). Pese a la fuerte crítica de Freud y parte del campo psicoanalítico, que veían en estas propuestas un regreso a las viejas teorías traumáticas, Ferenczi pudo leer su trabajo.

Reich, que ve entre los teóricos de la teoría de la libido y los partidarios de la pulsión de muerte una brecha infranqueable (Frigola, jul. 2015, p.6), resulta uno de los opositores más claros de la pulsión de muerte, tal y como da cuenta su artículo “El carácter masoquista: una refutación sexo-económica del instinto de muerte y la compulsión a la repetición” ( Reich, 1932 ).

Frigola considera la posición de Reich dentro del movimiento psicoanalítico como otro de los puntos de inflexión que supone el congreso de Lucerna, dado que su compromiso político se había ido revelando, cada vez más, como un peligro potencial. Desde 1933, solo se le permitiría supervisar a analistas miembros, y en secreto (Frigola, jul. 2015, p.15). En ese mismo año, se le comunicó que no podría publicar *Análisis de carácter* en la prensa analítica oficial, como sí venía ocurriendo con sus otras obras. Unos días antes del Congreso de Lucerna, y pese a ser uno de los ponentes, se le indica que su nombre ha tenido que ser eliminado de los registros de la Sociedad Psicoanalítica Alemana.

Para Frigola, este congreso signa el alejamiento del psicoanálisis de la sexualidad infantil y de los orígenes biológicos de la neurosis, lo que tiene consecuencias de calado. Considera “devastador” el hecho de que la psiquiatría no cuente con una psicoterapia psicoanalítica de base biológica, cuestión a la que hace responsable tanto de la hegemonía del enfoque farmacológico como del mecanicismo que lastra los tratamientos psicológicos (Frigola, jul. 2015, p.18). Por otro lado, hace una interesante analogía de la polémica entre los partidarios de la pulsión de muerte y los de la teoría de la libido durante los años 1930 con el movimiento psicoanalítico contemporáneo, al que entiende dividido en dos grupos diferenciados. Por un lado, quienes sostienen la primacía de las pulsiones y las fantasías en la génesis de la psique y, por tanto, consideran como tarea principal la decodificación de la fantasía y de los motivos inconscientes. Por otro, quienes entienden el desarrollo del ser humano un resultado de su experiencia relacional, y para quienes la tarea terapéutica esencial es analizar la experiencia y la re-experiencia de los modos de relación. Para Frigola, entender esto es esencial para quienes quieren escoger en qué asociaciones formarse en el campo psicodinámico.

Merece la pena señalar aquí que, en el contexto español, la configuración del psicoanálisis relacional es heredero, al menos parcialmente, de las propuestas de un psicoanálisis argentino politizado y con fuertes influencias marxistas ( Huertas-Maestro, 2021 ).

## Consideraciones finales

Como hemos intentado mostrar en las páginas previas, el freudomarxismo fue objeto de interés durante los años del tardofranquismo y de la Transición por parte de profesionales psi del ámbito territorial español. De esto da cuenta el trabajo realizado por Ramón García desde la editorial Anagrama para la divulgación y comprensión de un Wilhem Reich poco conocido en su época. La trayectoria de Carlos Frigola, quien tras formarse junto a Eva Reich fundaría la Fundación Wilhelm Reich, abierta aún en la actualidad, nos permite mostrar la dimensión que la recepción de Reich tuvo en la práctica asistencial de la época. Frigola también dedicó en años recientes varios artículos a la vida y obra de Reich, trazando interesantes analogías entre la situación del psicoanálisis de entreguerras y el actual. El freudomarxismo también recibió la atención de parte del ámbito académico como Antonio Caparrós i Benedicto, y también de psicoanalistas de relevancia para el proceso de politización del psicoanálisis argentino como Antonio Caparrós García-Moreno y José Bleger, los cuales serían asimismo importantes para configurar las influencias sociales y relacionales de una parte del psicoanálisis español.

Pese a las condiciones de un contexto español politizado por las movilizaciones del fin del franquismo y de la Transición, y ávido de herramientas de crítica social como podía ser el psicoanálisis, la difusión del freudomarxismo y, sobre todo, su arraigo, fue relativamente menor. Respecto a esto, podemos apuntar varias posibles explicaciones.

En primer lugar, las dificultades de los autores freudomarxistas para obtener un espacio de credibilidad por parte tanto de las instituciones psicoanalíticas como del movimiento comunista, una cuestión relevante en un contexto europeo marcado por la Guerra Fría y la aún potente hegemonía política del marxismo. En segundo, podría argumentarse que este nicho crítico, con voluntad de explorar la prometedora combinación de psicoanálisis y marxismo, fue ocupado por aquellos analistas que buscaron leer las teorías psicodinámicas desde el materialismo dialéctico, como es el caso de Bleger, Caparrós García-Moreno, y otras figuras del movimiento psicoanalítico argentino que ejerció su influencia sobre el contexto psi español. Además, y con la excepción de un Erich Fromm relevante para la construcción del psicoanálisis cultural y relacional estadounidense, la limitada circulación de las obras freudomarxistas en España pudo suponer un obstáculo esencial en este proceso. Merece la pena volver a señalar aquí el difícil estudio de la obra de Reich, no solo marcado por las limitaciones del fin del franquismo, sino también por las condiciones que él mismo puso al abordaje de su propia obra, y que hicieron que parte de ella no empezase a editarse hasta cincuenta años después de su muerte.

Aun quedando por valorar la influencia y los usos del freudomarxismo en otros ámbitos de la cultura y la crítica política ( Blanch, 1979 ), podríamos concluir que, al menos en el terreno profesional de la salud mental, la recepción del freudomarxismo en España fue más bien limitada, a pesar de los esfuerzos de destacadas figuras y de un contexto sociopolítico relativamente propicio, aunque complejo, para su desarrollo.
